# Melatonin Increases the Chilling Tolerance of Chloroplast in Cucumber Seedlings by Regulating Photosynthetic Electron Flux and the Ascorbate-Glutathione Cycle

**DOI:** 10.3389/fpls.2016.01814

**Published:** 2016-12-06

**Authors:** Hailiang Zhao, Lin Ye, Yuping Wang, Xiaoting Zhou, Junwei Yang, Jiawei Wang, Kai Cao, Zhirong Zou

**Affiliations:** ^1^College of Horticulture, Northwest A&F UniversityYangling, China; ^2^Key Laboratory of Protected Horticultural Engineering in Northwest, Ministry of AgricultureYangling, China; ^3^State Key Laboratory of Crop Stress Biology for Arid AreasYangling, China; ^4^College of Agricultural, Ningxia University, YinchuanNingxia, China; ^5^Department of Garden Engineering, Gansu Agriculture Technology CollegeLanzhou, China

**Keywords:** melatonin, chilling, chloroplast, ascorbate-glutathione cycle, photosynthetic electron flow

## Abstract

The aim of the study was to monitor the effects of exogenous melatonin on cucumber (*Cucumis sativus L.*) chloroplasts and explore the mechanisms through which it mitigates chilling stress. Under chilling stress, chloroplast structure was seriously damaged as a result of over-accumulation of reactive oxygen species (ROS), as evidenced by the high levels of superoxide anion ($O_{2}^{-}$) and hydrogen peroxide (H_2_O_2_). However, pretreatment with 200 μM melatonin effectively mitigated this by suppressing the levels of ROS in chloroplasts. On the one hand, melatonin enhanced the scavenging ability of ROS by stimulating the ascorbate–glutathione (AsA–GSH) cycle in chloroplasts. The application of melatonin led to high levels of AsA and GSH, and increased the activity of total superoxide dismutase (SOD, EC 1.15.1.1), ascorbate peroxidase (APX, EC 1.11.1.11), monodehydroascorbate reductase (MDHAR, EC 1.6.5.4) dehydroascorbate reductase (DHAR, EC 1.5.5.1), glutathione reductase (GR, EC1.6.4.2) in the AsA–GSH cycle. On the other hand, melatonin lessened the production of ROS in chloroplasts by balancing the distribution of photosynthetic electron flux. Melatonin helped maintain a high level of electron flux in the PCR cycle [***Je***(PCR)] and in the PCO cycle [***Je***(PCO)], and suppressed the O_2_-dependent alternative electron flux ***Ja***(O_2_-dependent) which is one important ROS source. Results indicate that melatonin increased the chilling tolerance of chloroplast in cucumber seedlings by accelerating the AsA–GSH cycle to enhance ROS scavenging ability and by balancing the distribution of photosynthetic electron flux so as to suppress ROS production.

## Introduction

Chilling represents as a very common abiotic stress to greenhouse-grown plants. Chilling-sensitive species, such as cucumber (*Cucumis sativus L.*), can be severely damaged by exposure to chilling temperatures as a result of loss of membrane integrity and associated reductions in enzyme activity (Heidarvand and Amiri, 2010). At low temperatures, the rates of biological reactions, particular of carbon dioxide reduction, are strongly reduced and this slowing limits the sinks for absorbed excitation energy (Allen and Ort, 2001). Therefore, even low irradiances can result in severe photoinhibition at low temperatures (Zhang et al., 2011). Photoinhibition not only decreases the photosynthetic ratio but also induces production of reactive oxygen species (ROS), and these lead to oxygen stress. Membranes can be damaged by excessive ROS, resulting in cell dysfunction such as the depression of membrane transport function and the membrane-associated enzyme activity (Zhan et al., 2014; Zhang et al., 2015a). To enhance plant tolerance to chilling and thus improve crop productivity, application of exogenous substances has been used widely (Shuming et al., 2012; Yang et al., 2015; Han et al., 2016). Transgenic approaches have also been used to achieve the same end (Sato et al., 2011; Bao et al., 2015). One of the exogenous substances trialed is melatonin, and this has been widely used to help organisms cope with stress, including with chilling stress (Zhang et al., 2015b).

Melatonin (*N*-acety-5-methoxytryptamine) is an indoleamine molecule. It was first found in plants in Dubbels et al. (1995) and Hattori et al. (1995). Since then, a wide range of species containing melatonin have been identified, indeed melatonin has been found in nearly all plant organs and tissues examined (Dubbels et al., 1995; Hattori et al., 1995; Manchester et al., 2000; Burkhardt et al., 2001; Chen et al., 2003; Hernández-Ruiz et al., 2004; Afreen et al., 2006; Tan et al., 2007a; Murch et al., 2010; Zhao et al., 2011; Arnao and Hernández-Ruiz, 2013, 2014; Shi et al., 2015). In addition to being a growth promoter and rooting agent (Sarrou et al., 2014), melatonin plays an important role in protecting plants from abiotic stress (Arnao and Hernández-Ruiz, 2015; Reiter et al., 2015), including from: UV radiation (Arnao and Hernández-Ruiz, 2014), heavy metals (Tan et al., 2007b), extreme temperatures (Bajwa et al., 2014) and salinity (Li et al., 2012). All these stresses lead to over-production of ROS (Zhang et al., 2015b). Melatonin itself is an effective antioxidant, which directly scavenges ROS and whose metabolite, *N1*-acetyl-*N2*-formyl-5-methoxykynuramine (AMFK), can also directly and efficiently scavenge ROS (Tan et al., 2007a; Manchester et al., 2015). Beyond this, melatonin also possesses antioxidant activity operating by stimulating antioxidant enzymes and augmenting antioxidants and involving the ascorbate–glutathione (AsA–GSH) cycle to scavenge excess ROS (Wang et al., 2012; Li et al., 2015; Chao et al., 2016). However, previous studies on the effects of melatonin on the AsA–GSH cycle under stress conditions are based mainly on whole leaves. Occurrence of ROS has also been reported in cell organelles, in chloroplasts, mitochondria and peroxisomes, and in the cytoplasm (Palma et al., 2006). The effects of melatonin on the AsA–GSH cycle may be different in different parts of a cell. Chloroplasts, being the site of photosynthesis, are also the primary source of ROS. The mechanisms through which melatonin affects the AsA–GSH cycle in chloroplasts are still not clear.

**FIGURE 1:**
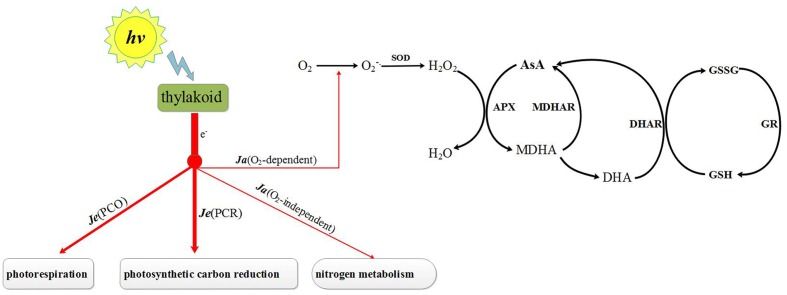
**Photosynthesis electron fluxes and AsA–GSH cycle in chloroplast.** *Je*(PCR), electron fluxes associated with photosynthetic carbon reduction; *Je*(PCO), electron fluxes associated with photorespiration; *Ja*(O_2_-independent), O_2_-independent alternative electron fluxes; *Ja*(O_2_-dependent), O_2_-dependent alternative electron fluxes; SOD, superoxide dismutase; APX, ascorbate peroxidase; MDHAR, monodehydroascorbate reductase; DHAR, dehydroascorbate reductase; GR, glutathione reductase; AsA, ascorbate acid; MDHA, monodehydroascorbate; DHA, reduced ascorbate acid; GSH, glutathione; GSSG, oxidized glutathione.

The level of ROS in chloroplasts is not only dependent on ROS scavenging ability but also on the ROS generating rate (**Figure 1**). The generation of ROS in chloroplasts is affected mainly by the distribution of electron fluxes through photosystem II (Zhou et al., 2004). The electron fluxes can be categorized into three groups: (1) electron fluxes associated with photosynthetic carbon reduction [***Je***(PCR)], (2) electron fluxes associated with photorespiration [***Je***(PCO)] and (3) alternative electron fluxes (***Ja***) (Miyake and Yokota, 2000; Jiang et al., 2013). One of the last of these is O_2_-independent [***Ja***(O_2_-independent)] and the other is O_2_-dependent [***Ja***(O_2_-dependent)] (Miyake and Yokota, 2000, 2001; Peeva et al., 2012). ***Ja***(O_2_-dependent) is one important source of ROS and its increase will led to the ebullition of ROS under stress condition (Zhou et al., 2004),. Under stress, ***Ja***(O_2_-dependent) fluxes increased dramatically along with a reduction in ***Je***(PCR) (Liu et al., 2012). It has been reported that ***Ja***(O_2_-dependent) fluxes can be suppressed by phytohormones such as by brassinosteroids (Jiang et al., 2013). These are similar to one type of phytohormone but whether melatonin has the same function is unknown. Earlier studies show that melatonin can regulate the expressions of the photosystem I genes *PsaA, PsaF, PsaG, PsaH, PsaK* and *PsaO*, of the photosystem II genes *PsbE, PsbO, PsbP, PsbQ*, *PsbY, PsbZ* and *Psb28*, and of the Calvin cycle genes *rbcS, GAPC1 and GAPCP-2* (Wei et al., 2014). The variation of photosynthesis genes expression may led to changes of the electron fluxes in PSII. Therefore, the role of melatonin in regulating the distribution of electron fluxes in PSII under stress conditions is also worth exploring.

In this study, we determine whether melatonin pretreatment can alleviate chilling stress by regulating ROS levels in chloroplasts of cucumber. As well as exploring the mechanisms through which melatonin affects the AsA–GSH cycle (an important antioxidant system scavenging ROS in chloroplasts) we also explored the role of melatonin in regulating the distribution of electron fluxes in photosystem II (which is largely responsible for the production of ROS in chloroplasts).

## Materials and Methods

### Plant Material and Treatments

Seeds of cucumber (*Cucumis sativus* L.) cultivar Xinyan 4 were planted singly in black plastic pots (7 cm × 7 cm) filled with mixed nutrient medium (peat: vermiculite: perlite = 2:1:1) and placed in a growth chamber under standard conditions [28/18°C day/night temperatures, 12 h photoperiod, 300 μmolm^-2^ s^-1^ photon flux density and 75% relative humidity (RH)]. From the time the second leaf was fully expanded, the nutrient medium of half the seedlings was irrigated with 10 mL 200 μM melatonin solution at eight o’clock every night. 0.1856 g melatonin was firstly solved in 4 mL ethanol and then dilute with water to 4 L to get 200 μM melatonin – this concentration had been determined in a previous trail (unpublished data) to be quite effective in lowering the ROS level. The rest were irrigated with 10 mL distilled water contain the same ethanol concentration until the experiment was terminated every day. After 5 days of melatonin treatment, the groups of water- and melatonin-treated seedlings were each randomly divided into two subgroups. One subgroup of each was moved to another growth chamber with a chilling environment (15/8°C day/night temperatures, 12 h photoperiod, 300 μmolm^-2^ s^-1^ photon flux density and 75% RH) while the other two subgroups were grown on under the former standard conditions. This resulted in four different subgroups: (i) *Control* – grown under standard conditions and irrigated with distilled water, (ii) *Control + Melatonin* – grown under standard conditions and irrigated with melatonin, (iii) *Chilling* – grown under chilling conditions and irrigated with distilled water, and (iv) *Chilling + Melatonin* – grown under chilling conditions and irrigated with melatonin.

Leaf samples were taken from all four subgroups (i, ii, iii, iv), 0, 2, 4, 6, and 8 days after the chilling treatment was imposed (on subgroups iii and iv). These were rapidly frozen in liquid nitrogen and stored at -80°C pending analysis. Photosynthesis and fluorescence parameters were measured on leaves of all subgroups 4 days after chilling treatment.

### Isolation of Chloroplasts

Intact chloroplasts were isolated according to Robinson et al. (1983) with some modification. Leaves (10 g) were extracted at 0°C with 20 mL of isolation buffer containing 330 mM sorbitol, 30 mM Mes, 2 mM ascorbate and 0.1% BSA adjusted to pH 6.5 with Tris. The brei was filtered through four layers of cotton wool and centrifuged at 1200 g for 3 min. After discarding the supernatant, the pellets were re-suspended in 2 mL of suspension buffer containing 330 mM sorbitol, 30 mM Hepes and 0.2% BSA adjusted to pH 7.6 with Tris. The suspension was placed in centrifuge tubes containing 3.5 mL of 80% percoll and 3.5 mL of 40% percoll, and then centrifuged at 1200 g for 1 min. Intact chloroplasts were found in the intermediate region between 80% percoll and 40% percoll.

### Analysis of Malonaldehyde (MDA), Superoxide Anion ($O_{2}^{-}$) and Hydrogen Peroxide (H_2_O_2_) in Chloroplasts

For the MDA assay, 1 mL of chloroplast suspension was homogenized with 2 mL of 10% TCA and centrifuged at 4000 *g* for 10 min. Next, 700 μL of the supernatant was incubated at 100°C in a boiling water bath for 15 min with 700 μL of 0.6% thiobarbituric acid (TBA) dissolved in 10% TCA. The absorptions were measured at 600, 532, and 450 nm. The content of MDA was calculated as described by Heath (Heath and Packer, 1968).

The $O_{2}^{-}$ was assayed as described by Ke et al. (2007) with some modification. First, 675 μL of chloroplast suspension was homogenized with 675 μL of phosphate buffer (pH 7.8) and 1 mL hydroxylamine hydrochloride. The mixture was incubated at 25°C for 20 min. Then, 0.375 mL of 17 mM γ-amino-phenylsulfonic and 0.375 mL of 7 mM α-amino-phenylsulfonic were added to the mixture for another 20 min of incubation at 30°C. Next, 3.2 mL of ether was added to avoid interference from chlorophyll. The absorption of the reaction mixture was measured at 530 nm. $O_{2}^{-}$ was calculated from a standard curve based on sodium nitrite.

Hydrogen peroxide (H_2_O_2_) was assayed as described by Sergiev et al. (1997) with some modification. First, 1 mL of chloroplast suspension was homogenized with 2 mL of ice-cold 0.1% TCA and centrifuged at 12,000 *g* for 15 min. Then, 0.5 mL of the supernatant was added to 0.5 mL of 100 mM potassium phosphate buffer (pH 7.0) and 1 mL of 1 M KI and incubated for 1 h in darkness. Absorbance was measured at 390 nm. The content of H_2_O_2_ was calculated base a standard curve.

To detect H2O2 production in chloroplast, chloroplasts were incubated in 5 μM of 2′,7′-dichlorofluorescein diacetate (DCFH-DA) dissolved in chloroplast suspension buffer for 30 min at 25°C under darkness. After washing three times, green fluorescence, indicative of the oxidation by H_2_O_2_ of DCFH-DA to 2′,7′-dichlorofluorescin (DCF), was monitored (excitation: 488 nm, barrier: 500–550 nm) in a FV1200 confocal laser scanning microscope (OLYMPUS, Japan).

### Analysis of Antioxidant Metabolites in Chloroplasts

A volume of 300 μL of chloroplast suspension was homogenized with 1.2 mL of ice-cold 6% (v/v) HClO_4_ and centrifuged at 4°C for 10 min at 10,000 *g*. The supernatant was used to assay AsA and reduced ascorbate acid (DHA) as described by Wang et al. (2012).

First, 300 μL of chloroplast suspension was homogenized with 1.2 mL of 5% sulfosalicylic and centrifuged at 4°C for 10 min at 14,000 *g*. The supernatant was used to assay glutathione (GSH) and oxidized glutathione (GSSG) as described by Wang et al. (2012).

### Analysis of Antioxidant Enzymes in Chloroplasts

A volume of 3 mL of chloroplast suspension was homogenized with 3 mL of ice-cold 25 mM Hepes buffer (pH 7.8) containing 0.2 mM EDTA and 2% PVP and centrifuged at 4°C for 10 min at 13,000 *g*. The supernatant was used to analyze antioxidant enzyme in chloroplasts.

Total SOD (EC 1.15.1.1) was measured as described by Thayer (1990). One unit of SOD was defined as the amount of enzyme inhibiting 50% of NBT reduction.

For ascorbate peroxidase (APX, EC 1.11.1.11), the decrease in absorbance at 290 nm was measured as a result of the oxidation of reduced ascorbate (ASC) (extinction coefficient of 2.8 mM⋅cm^-1^). The reaction mixture (3 mL) contained 50 mM Hepes-KOH (pH 7.6), 0.1 mM EDTA-Na_2_, 0.5 mM ASC, 0.2 mM H_2_O_2_, and 100 μL enzyme extract. The reaction was initiated by adding H_2_O_2_ (Logan et al., 1998).

For monodehydroascorbate reductase (MDHAR, EC 1.6.5.4), the decrease in absorbance at 340 nm was measured following the oxidation of NADH (nicotinamide adenine dinucleotide, reduced) (extinction coefficient of 6.22 mMcm^-1^). The reaction mixture (3 mL) contained 50 mmol⋅L^-1^ K-phosphate buffer (pH 7.3), 0.2 mmol⋅L^-1^ NADH, 1.0 mmol⋅L^-1^ ascorbate, 1.0 unit of ascorbate oxidase, and 100 μL enzyme extract. The reaction was initiated by adding ascorbate oxidase (Erkan et al., 2008).

For dehydroascorbate reductase (DHAR, EC 1.5.5.1), the increase in absorbance at 265 nm was measured as a result of ASC formation (extinction coefficient of 14 mM⋅cm^-1^). The reaction mixture (3 mL) contained 100 mM Hepes-KOH (pH 7.0), 1 mM EDTA, 2.5 mM reduced glutathione (GSH), 0.2 mM dehydroascorbate (DAsA) and 100 μL enzyme extract. The reaction was initiated by adding DAsA (Dalton et al., 1986).

For GR (EC1.6.4.2), the decrease in absorbance at 340 nm was measured as a result of NADPH oxidation (extinction coefficient of 6.22 mM⋅cm^-1^). The reaction mixture (3 mL) contained 100 mM Tris-HCl (pH 8.0), 1 mM EDTA, 1 mM oxidized glutathione (GSSG), 0.2 mM NADPH, and 100 μL enzyme extract. The reaction was initiated by adding NADPH (Grace and Logan, 1996).

### RNA Isolation and Quantitative Real-Time RT-PCR

Total RNA was extracted from leaves with a plant RNA kit (Omega Bio-Tek, Doraville, GA, USA) and then reverse-transcribed using a PrimeScript^TM^ RT reagent kit with gDNA Eraser (Takara, Shiga, Japan) according to the manufacture′s instruction. Real-time PCR was carried out on a StepOne^TM^ Plus real-time PCR system (Applied Biosystems, USA) using a SYBR Premix EX Taq kit (Taraka). The specific primers (**Supplementary Table S1**) used in the present study were designed using Primer Premier 6 software (Biosoft International, Palo Alto, CA, USA). The actin gene acted as the internal standard. Relative fold expression changes were calculated using the (2^-ΔΔC^_t_) method.

### Transmission Electron Microscope (TEM) Analyses

Small pieces (∼1 mm^2^) cut from the same region of different leaves were fixed with 4% glutaradehyde in 0.2 mM sodium phosphate buffer (pH 6.8) for 6 h at 4°C. After washing with 0.1 M sodium phosphate buffer (pH 6.8), the tissue pieces were post-fixed for 2.5 h in 1% osmic acid in 0.2 mM sodium phosphate buffer (pH 6.8) and re-washed with 0.1 M sodium phosphate buffer (pH 6.8). Following dehydration in a graded series of ethanol solutions, the tissue pieces were embedded in Epon-812 and sectioned using an ultramicrotome (EM UC7, Leica Microsystems GmbH, Germany). A TEM (JEM-1230, JEOL, Japan) was used to assay the ultrathin sections stained with uranyl acetate and lead citrate.

### Leaf Gas Exchange and Chlorophyll Fluorescence Analyses

For leaf gas exchange and chlorophyll fluorescence analysis, plants were placed in a growth chamber at 25°C and held for 1 h at a photon flux density of 600 μmol⋅m^-2^⋅s^-1^ before measurement. Leaf gas exchange and chlorophyll fluorescence parameters were measured simultaneously using a portable photosynthesis system (LI-6400XT, LI-COR, USA) equipped with a leaf chamber fluorimeter (LI6400-40, LI-COR, USA) under both photorespiratory (21% O_2_) and non-photorespiratory (2% O_2_) conditions (Zhou et al., 2004). The air temperature, CO_2_ concentration and photosynthetic photon flux intensity (PPFD) were set at 25°C, 350 μmol⋅mol^-1^, 600 μmol⋅m^-2^⋅s^-1^. The A-Ci curves under PPFD 300 and 600 μmol⋅m^-2^⋅s^-1^ were measured between 0 and 1200 μmol⋅mol^-1^ CO_2_ at 21% O_2_ and 25°C (Brooks and Farquhar, 1985).

### Estimation of the Rate of Electron Flux

The rate of electron transport through PSII [***Je***(PSII)] is equal to α × Φ(PSII) × PPFD where α is the absorbance coefficient ratio of allocation of excitation energy to PSIIwhich can be expressed as 4 × (A+Rd)/[PFD × Φ(PSII)] under non-photorespiratory conditions (Genty et al., 1989; Miyake and Yokota, 2000). The rate of carboxylation by Rubisco (Vc) was calculated according to the description of Miyake [25], and the rate of oxygenation by Rubisco (Vo) following Von Caemmerer and Farquhar (1981; Miyake and Yokota, 2000). The electron flux in the PCR cycle [***Je***(PCR)] equals 4 × Vc while the electron flux in the PCO cycle [***Je***(PCO)] is equal to 4 × Vo (Krall and Edwards, 1992). The alternative flux (***Ja***) can be expressed as ***Je***(PSII)-***Je***(PCR-***Je***(PCO) (Miyake and Yokota, 2000). The $O_{2}^{-}$ dependent alternative electron flux ***Ja***($O_{2}^{-}$dependent) equals***Ja***(21%O_2_)- ***Ja***(2%O_2_) (Miyake and Yokota, 2000).

### Statistical Analyses

The data are expressed as the means ± standard deviations of three replicate samples. One way ANOVA was used to analyze all data followed by Duncan’s multiple range tests. A probability of *P* < 0.05 was considered statistically significant.

## Results

### Ultrastructural Changes in Chloroplasts

**FIGURE 2:**
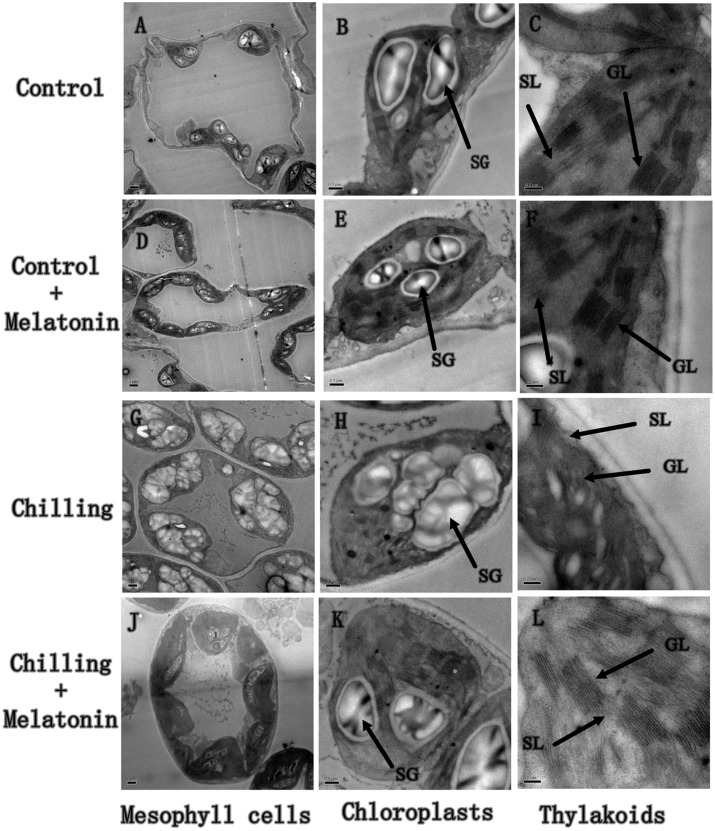
**Effects of exogenous melatonin on chloroplast structure under chilling stress.** The second, fully expanded leaves were measured after 4 days of chilling. **(A–C)** *Control* (28/18°C); **(D–F)** *Control + Melatonin* (200 μM melatonin, 28/18°C); **(G–I)** *Chilling* (15/8°C); **(J–L)** *Chilling + Melatonin* (200 μM melatonin, 15/8°C). SL, stoma lamellae; GL, grana lamellae; SG, starch grains.

To examine whether melatonin could alleviate damage to chloroplasts induced by chilling, we investigated chloroplast ultrastructure. Under standard temperature conditions, chloroplasts were attached to the cell wall and exhibited typical ellipsoidal shapes. Regularly arranged grana lamellae were interconnected by well-developed stromal lamellae (**Figures 2A–F**). Under chilling stress, the chloroplasts became swollen and contained more starch grains and the lamella system was also swollen and indistinct (**Figures 2G–I**). Application of exogenous melatonin significantly reduced chloroplast damage indicated by a more normal chloroplast ultrastructure (**Figures 2J–L**).

### MDA Contents and ROS Levels

**FIGURE 3:**
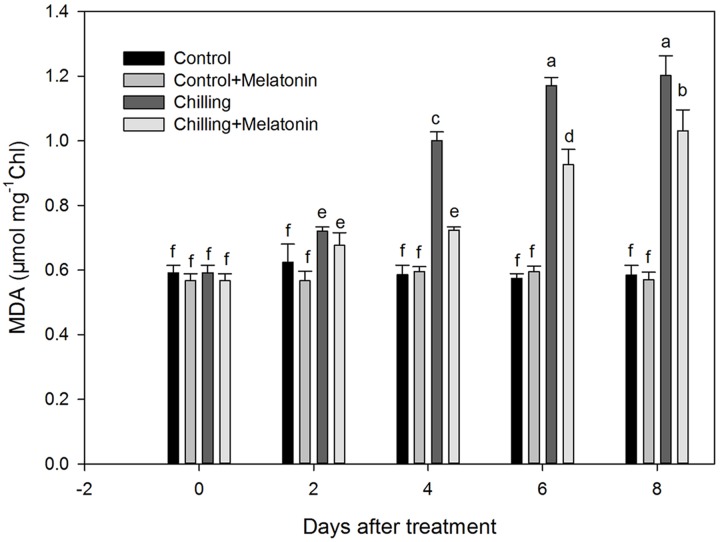
**Effects of exogenous melatonin on malonaldehyde (MDA) content of chloroplasts under chilling stress.** *Control* (28/18°C); *Control + Melatonin* (200 μM melatonin, 28/18°C); *Chilling* (15/8°C); *Chilling + Melatonin* (200 μM melatonin, 15/8°C). Data are means ± SD of three replicates. Different letters indicate significant differences according to a Duncan’s multiple range test (*P* < 0.05).

Further, we monitored chloroplast MDA content. Our data confirm that chilling induced dramatic accumulations of MDA in chloroplasts (**Figure 3**). Between day 2 and day 6, the MDA content rose sharply, followed by a further slight increase over the next 2 days. However, exogenous melatonin application not only delayed the rise by 2 days but also weakened it.

**FIGURE 4:**
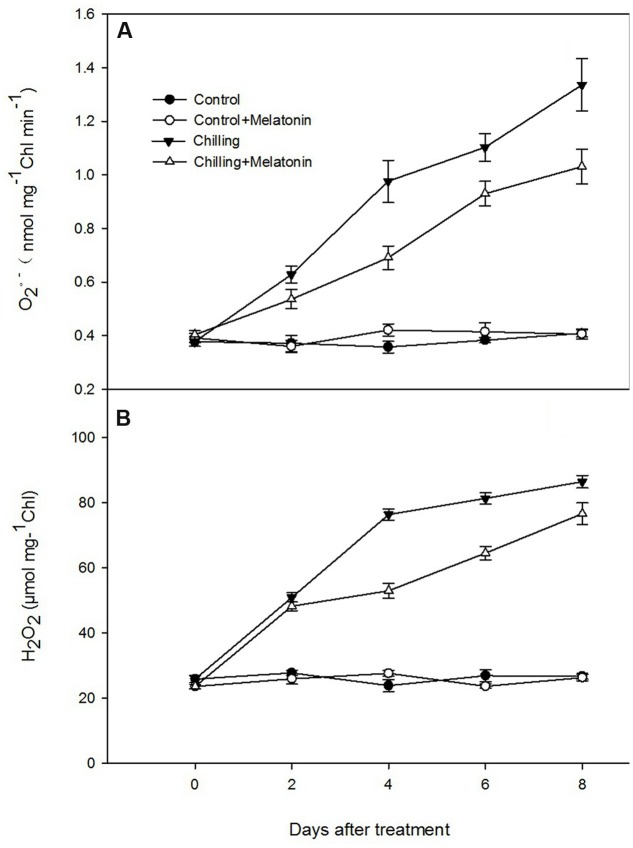
**Effects of exogenous melatonin on** **(A)** superoxide anion (O_2_-) and **(B)** hydrogen peroxide (H_2_O_2_) contents of chloroplasts under chilling stress. *Control* (28/18°C); *Control + Melatonin* (200 μM melatonin, 28/18°C); *Chilling* (15/8°C); *Chilling + Melatonin* (200 μM melatonin, 15/8°C). Data are means ± SD of three replicates.

**FIGURE 5:**
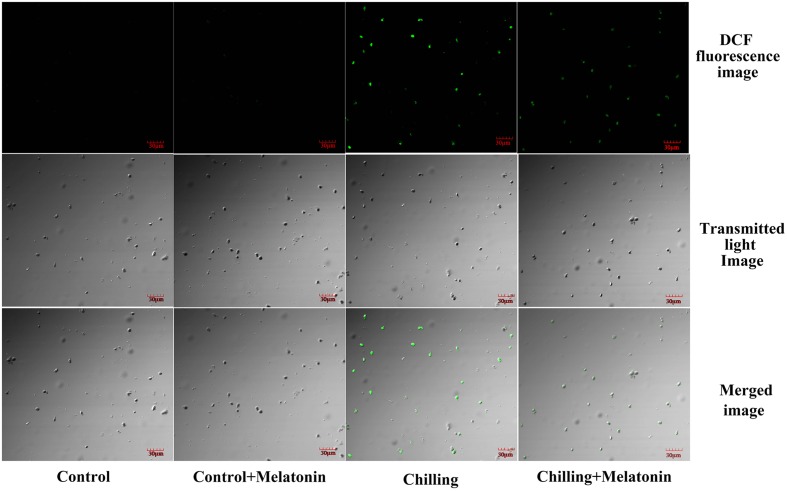
**Confocal laser scanning micrographs of DCFH-infiltrated chloroplast.** The second, fully expanded leaves were measured after 4 days of chilling. Control (28/18°C); *Control* + *Melatonin* (200 μM melatonin, 28/18°C); Chilling (15/8°C); *Chilling* + *Melatonin* (200 μM melatonin, 15/8°C). DCF fluorescence, indicative of H_2_O_2_; Transmitted light image, indicative of the number of chloroplasts; Merged image, combination of DCF fluorescence image and Transmitted light image.

The rate of generation of $O_{2}^{-}$ increased sharply during chilling while melatonin significantly reduced this increase especially at days 4 and 8 (**Figure 4A**). During the first 4 days, H_2_O_2_ levels rose dramatically and then increased slowly over the following days (**Figure 4B**). After 2 days, melatonin markedly suppressed the strong production of H_2_O_2_ and the inhibition gradually subsided as time passed. Those results were corroborated by observation of H_2_O_2_ production, seen as epifluorescence from chloroplast infiltrated with DCFH-DA. Chilling treatment present the strongest fluorescence while the fluorescence intensity of Chilling + Melatonin treatment was weaker than that of chilling treatment (**Figure 5**). Under non-stress conditions, And, $O_{2}^{-}$ and H_2_O_2_ levels in chloroplasts were little affected by melatonin.

### AsA–GSH Cycle

**FIGURE 6:**
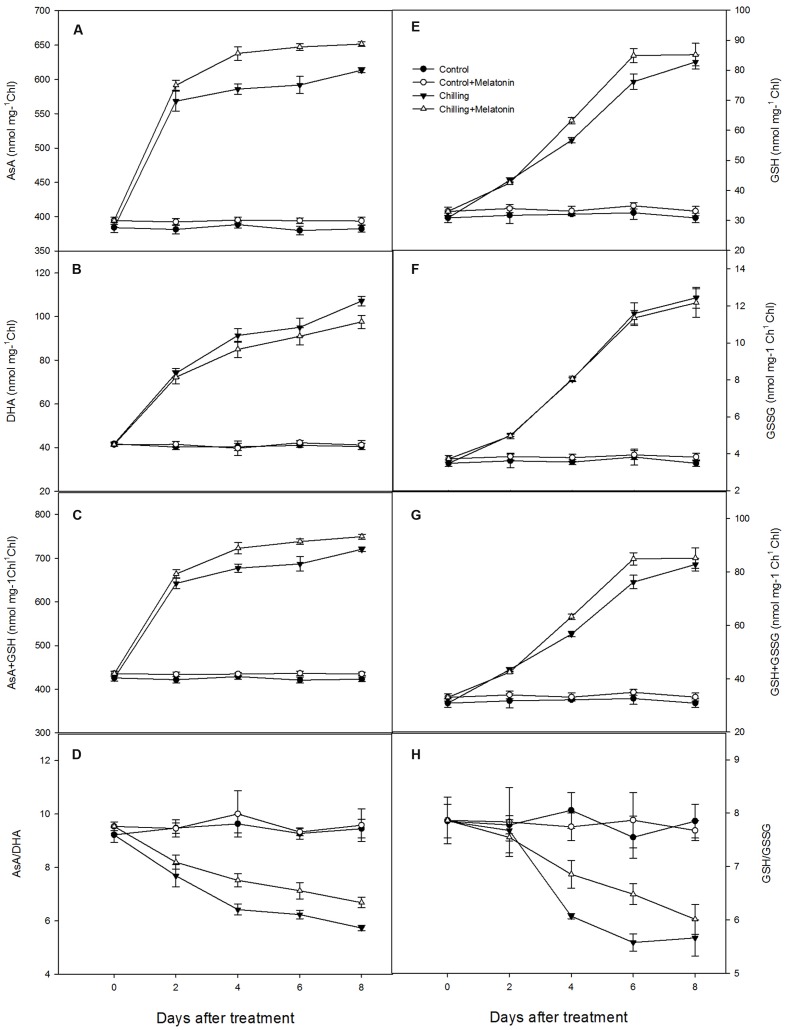
**Effects of exogenous melatonin on antioxidant content of chloroplasts under chilling stress:** **(A)** ascorbic acid (AsA), **(B)** dehydroascorbate (DHA), **(C)** AsA + DHA, **(D)** AsA/DHA, **(E)** glutathione (GSH), **(F)** oxidized glutathione (GSSG), **(G)** GSH + GSSG, **(H)** GSH/GSSG. Control (28/18°C); *Control* + *Melatonin* (200 μM melatonin, 28/18°C); Chilling (15/8°C); *Chilling* + *Melatonin* (200 μM melatonin, 15/8°C). Data are means ± SD of three replicates.

The interconversions between oxidized and reduced ascorbic and glutathione in chloroplasts were also investigated. Under chilling stress, the contents of AsA, and total AsA both increased dramatically over the first 2 days and then continued to climb more slowly over the following 6 days. This pattern was similar but slightly stronger in the melatonin-treated plants (**Figures 6A,C**). Chilling stress also led to a dramatic increase in DHA content but melatonin did not lead to any additional change except at day 8 (**Figure 6B**). Melatonin also led no change on GSSG content (**Figure 6F**). Levels of AsA/DHA decreased markedly, induced by chilling but were clearly mitigated by melatonin (**Figure 6D**). Meanwhile, levels of GSH/GSSG followed a similar pattern (**Figure 6H**). Chilling stress resulted in a sharp increase in the contents of GSH and total GSH over the first 6 days with the increase being reinforced by melatonin treatment – especially on days 4 and 6 (**Figures 6E,G**).

**FIGURE 7:**
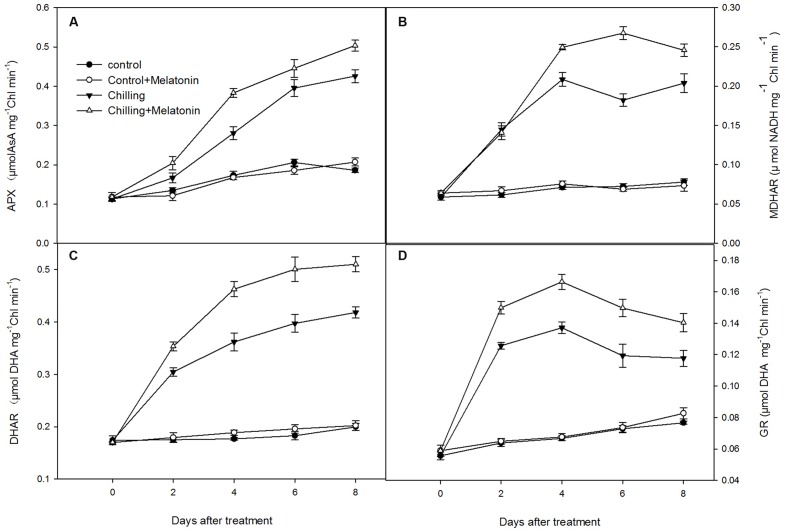
**Effects of exogenous melatonin on activities of key enzymes in ascorbic acid – glutathione (AsA–GSH) cycle in chloroplasts under chilling stress.** **(A)** ascorbate peroxidase (APX), **(B)** monodehydroascorbate reductase (MDHAR), **(C)** dehydroascobate reductase (DHAR), **(D)** glutathione reductase (GR). *Control* (28/18°C); *Control + Melatonin* (200 μM melatonin, 28/18°C); *Chilling* (15/8°C); *Chilling + Melatonin* (200 μM melatonin, 15/8°C). Data are means ± SD of three replicates.

Chilling stress dramatically increased the activities of the chloroplast antioxidant enzymes. Thus, under chilling, SOD activity increased dramatically over the first 2 days, before peaking on day 2 and then decreasing over the following days (**Supplementary Figure S1**). However, melatonin significantly delayed this later decrease. The activities of APX and DHAR climbed rapidly during chilling, and this trend was strengthened by melatonin (**Figures 7A,C**). The activities of MDHAR and GR increased with chilling until day 4 and then fluctuated over the following few days (**Figures 7B,D**). Melatonin clearly increased the activities of MDHAR and GR.

**FIGURE 8:**
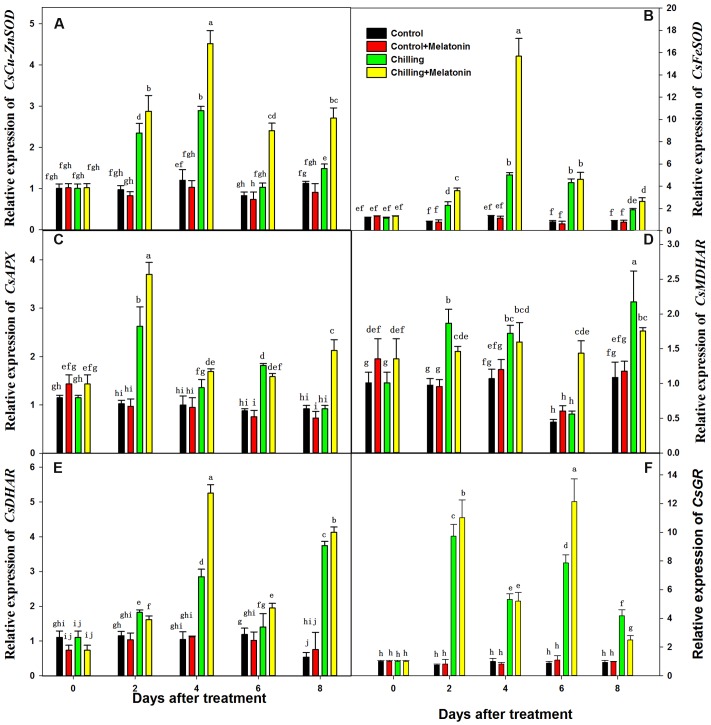
**Effects of exogenous melatonin on transcript levels of key genes involved in biosynthesis of enzymes of ASA-GSH cycle under chilling stress.** **(A)** *CsCu-ZnSOD*, **(B)** *CsFeSOD*, **(C)** *CsAPX*, **(D)** *CsMDHAR*, **(E)** *CsDHAR*, **(F)** *CsGR. Control* (28/18°C); *Control + Melatonin* (200 μM melatonin, 28/18°C); *Chilling* (15/8°C); *Chilling + Melatonin* (200 μM melatonin, 15/8°C). Data are means ± SD of three replicates.

Expression levels of genes involved in the biosynthesis of these enzymes of ASA–GSH cycle were evaluated at the transcriptional level. The expression levels of *CsCu-ZnSOD*, *CsFeSOD*, *CsAPX*, *CsMDHAR*, *CsDHAR* and *CsGR* were significantly upregulated by chilling from day 2 (**Figure 8**). Compared with that of chilling treatment, the expression of *CsCu-ZnSOD* was further upregulated by melatonin during the whole period while *CsFeSOD* was only upregulated on day 4. Moreover, melatonin significantly upregulated the expression level of *CsAPX* (on days 2 and 8), *CsDHAR* (on days 4 day 8) and *CsGR* (on days 2 and 6) compared with chilling treatment. And, the expression level of *CsMDHAR* was only upregulated by melatonin on day 6.

### Electric Flux in Photosystem

With chilling, ***Je***(PSII) and ***Je***(PCR) slumped by 43 and 71% while ***Je***(PCO) and ***Ja***(O_2_-independent) increased by 11 and 74%, compared with the controls (**Table 1**). Moreover, Ja and ***Ja***(O_2_-dependent) were between 3- and 8-times higher than in the controls. However, melatonin slightly decreased ***Je***(PSII) and ***Je***(PCR) and increased ***Ja*** and ***Ja***(O_2_-dependent) and increased the growth of ***Ja***(PCO) and ***Ja***(O_2_-independent). Under un-chilled conditions, melatonin had no significant effect on electron flows, though ***Je***(PCO) did increase by about 20%.

**Table 1:** Effects of exogenous melatonin on photosynthetic electron partitions after four days chilling stress.

Treatments	Je(PSII) μmolm^-2^s^-1^	Je(PCR) μmolm^-2^s^-1^	Je(PCO) μmolm^-2^s^-1^	Ja21% μmolm^-2^s^-1^	Ja(O_2_-independent) μmolm^-2^s^-1^	Ja(O_2_-dependent) μmolm^-2^s^-1^
Control	75.57 ± 0.86a	61.01 ± 0.77a	11.60 ± 0.79c	2.96 ± 0.41c	1.97 ± 0.20c	0.99 ± 0.22c
Melatonin	77.27 ± 2.86a	60.02 ± 1.88a	14.10 ± 0.90b	3.16 ± 0.16c	2.20 ± 0.16c	0.96 ± 0.12c
Chilling	43.35 ± 1.57c	17.8 ± 0.41c	12.85 ± 0.52bc	12.82 ± 0.90a	3.42 ± 0.28b	9.40 ± 1.12a
Chilling + Melatonin	52.97 ± 1.41b	24.51 ± 0.12b	18.32 ± 0.61a	10.14 ± 1.10b	4.41 ± 0.44a	5.73 ± 0.66b


**Control* (28/18°C); *Control + Melatonin* (200 μM melatonin, 28/18°C); *Chilling* (15/8°C); *Chilling + Melatonin* (200 μM melatonin, 15/8°C). Data are means ± SD of three replicates. Different letters indicate significant (*P* < 0.05) differences based on Duncan’s multiple range text.*

## Discussion

### Melatonin Alleviates Chilling Damage With Cucumber Chloroplasts

Cucumber is an important salad crop but it is very sensitive to chilling (Xu et al., 2008). Under chilling stress, chloroplasts, the primary site of the photosynthetic reactions, commonly present signs of membrane damage. This is because chloroplasts are a major source of ROS which cause membrane lipid peroxidation (Asada, 2006; Gill and Tuteja, 2010). The degree of membrane damage in chloroplasts can be seen in their ultrastructure (Yuan et al., 2012; Shu et al., 2013; Shao et al., 2014). Our results show that under chilling, the chloroplasts swell and alter their typical ellipsoidal shape (**Figures 2G–I**). Melatonin significantly alleviates these indications of chilling damage with their shape and structure appearing normal.

### Melatonin Reduces the ROS Levels and MDA Contents in Cucumber Chloroplasts

Accumulation of ROS such as $O_{2}^{-}$ and H_2_O_2_ occurred under chilling and was associated with signs of chloroplast damage. Although low levels of ROS are indispensable, excessive accumulations had been shown to be associated with damage from lipid peroxidation, and oxidation of protein and DNA (Gill and Tuteja, 2010; Heidarvand and Amiri, 2010; Xia et al., 2014). Lipid peroxidation from excessive ROS leads to structural abnormalities and cell dysfunction of (Gill and Tuteja, 2010). Malonaldehyde is usually considered an indicator of membrane structural integrity (Xi et al., 2013). In the present study, chloroplast MDA content increased dramatically (**Figure 3**) under chilling as a result of production of $O_{2}^{-}$ and H_2_O_2_ (**Figures 4** and **5**). However, with melatonin these negative indications were significantly reduced. This is consistent with the finding reported by Wang et al. (2016) in cucumber under salinity-induced stress. Thus it is clear that melatonin helps to reduce membrane damage caused by over-accumulation of ROS. Other studies have shown that exogenous melatonin can decrease ROS in plants exposed to salinity stress (Li et al., 2012), drought stress (Zhang et al., 2013) and during senescence (Wang et al., 2012). Consistent with these studies, our results confirm that melatonin can protect chloroplasts from ROS damage under chilling stress.

### Melatonin Regulates ROS Metabolism Through Both Their Generation and Their Elimination

The level of ROS in a chloroplast depends on the balance between the rate at which they are generated and that at which they are eliminated. Here, we explored the role of melatonin in regulating ROS metabolism – both their generation and their elimination in chloroplasts of chilling-stressed cucumbers (**Figure 1**).

An important and efficient ROS-scavenging pathway in chloroplasts is the AsA–GSH cycle (Palma et al., 2006). After $O_{2}^{-}$ has been reduced by SOD to H_2_O_2_, the excess levels of H_2_O_2_ are scavenged by transforming it to H_2_O under catalysis by APX using AsA as the electron donor. Meanwhile, AsA is oxidized to MDHA, which is then reduced directly to AsA by MDHAR or first degraded to DHA and then be reduced to AsA by DHAR using GSH as the reducing substrate. The oxidized substrate of GSH, GSSG, can be reduced to GSH by GR (Davey et al., 2000). Chilling stress leads to decreases in AsA/DHA and to increases in AsA, DHA and total AsA (**Figure 6**). The decrease in AsA/DHA shows a higher percentage of AsA is transformed to the oxidized state in response to increased oxidative stress. Moreover, melatonin increased the AsA content, but had no significant influence on the DHA content (**Figures 6A,B**). This indicates that melatonin may elevate the AsA sink by increasing AsA content and is also helpful in maintaining a high level of AsA/DHA. Similar founding appeared in GSH and GSSG (**Figures 6E,F**). Maintaining the efficient recycling of AsA via APX, MDHAR and DHAR is crucial to maintaining AsA in a high redox state so that it scavenges H_2_O_2_ efficiently (Conklin and Barth, 2004). Ascorbate is an important antioxidant in the AsA–GSH cycle and usually works in combination with GSH (Nagalakshmi and Prasad, 2001). Under chilling stress, the total AsA content rose to a high level at day 2 (**Figure 6C**) while the activity of APX, DHAR and MDHAR increased relatively slowly in the early period (**Figure 7**). This indicates that AsA biosynthesis plays a dominant role in maintaining a high ratio of AsA/GSH in the early period, while the efficient recycling of AsA in the later period was due mainly to the high activity of APX, DHAR and MDHAR. The key enzyme catalyzing the reduction from H_2_O_2_ to H_2_O is APX, and this uses AsA as electron donor while the recycling of AsA is dependent on MDHAR and DHAR (Wang et al., 2013). Melatonin has been shown to stimulate the activity of APX, DHAR and MDHAR(Wang et al., 2012). Ascorbate peroxidase is found in apple leaves under oxidative stress (Wang et al., 2012). This finding is in accordance with our results (**Figures 6A–C**). The recycling of AsA must also be coupled with the recycling of GSH catalyzed by GR. A high GSH/GSSG ratio helps the recycling of AsA by maintaining an appropriate redox environment and reducing oxidative stress (Wang et al., 2013). Studies in animals show that melatonin can stimulate γ-gulamylcysteine synthetase to preserve GSH levels in cells exposed to ROS (Urata et al., 1999). Melatonin can also accelerate GSH in apple leaves (Wang et al., 2012). Our findings are similar (**Figure 6D**). SOD can accelerate the reduction from $O_{2}^{-}$ to H_2_O_2_ which is an earlier reaction of AsA–GSH. Our results also show that melatonin helps to enhance SOD activity during chilling (**Supplementary Figure S1**). This finding is consistent with an earlier study in apple leaves under salt stress (Li et al., 2012). Our results demonstrate that melatonin activated the AsA–GSH cycle by enhancing the activity of SOD, APX, MDHAR, DHAR and GR, and by increasing the contents of AsA and GSH thus maintaining a high level of AsA/DHA and GSH/GSSG under chilling stress. Apart of that, we also found that the enhancement in activities of enzymes of AsA–GSH cycle was related the upregulation of genes involved in the biosynthesis of these enzymes of ASA–GSH cycle induced by melatonin. The study confirms that the AsA–GSH cycle plays an important role in mitigating the function of exogenous melatonin under chilling conditions. The AsA–GSH cycle, an important antioxidant system in higher plants, has been reported in chloroplasts, mitochondria, peroxisomes and cytoplasm of higher plants (Palma et al., 2006; Zhang et al., 2015b). Just how melatonin stimulates the AsA–GSH cycle is not clear, however, suggesting further investigation in this area would be worthwhile.

The generation of ROS in chloroplasts is closely correlated with the electron flux in the photosystem (Jiang et al., 2013). Under normal conditions, the photon energy absorbed by chlorophyll is mainly converted to chemical energy and stored in the form of NADPH and ATP through photosynthetic linear flow of electrons. In C3 plants, NADPH and ATP are the sources of energy in both the photosynthetic carbon-reduction (PCR) cycle and the photorespiration carbon-oxidation (PCO) cycle. Under stress, however, the consumption of NADPH and ATP falls below the rate of photosynthetic linear flow of electrons, on account of the reduced activity of the PCR and PCO cycles. After that, the photosynthetic linear flux of electrons [***Je***(PCR) and ***Je***(PCO)] is full and part of the excessive electron will flow into alternative electron flux depends on the partial pressure of O_2_ [***Ja***(O_2_-depend)], which will reduce O_2_ and lead to overexpression of ROS (Miyake and Yokota, 2000, 2001; Peeva et al., 2012). Under chilling stress, the O_2_-dependent alternative electron flux increases indicating a high proportion of electrons transported to O_2_ producing ROS (Zhou et al., 2004; Liu et al., 2012; Jiang et al., 2013). Consistent with our study, ***Ja***(O_2_-dependent) increased dramatically while ***Je***(PSII) and ***Je***(PCR) decreased sharply under chilling (**Table 1**). These behaviors imply that a considerable excess of energy was trapped which was then consumed through the alternative electron flux depending on the partial pressure of O_2_, this led to overproduction of ROS. In our experiments, melatonin not only maintained high levels of ***Je***(PSII), ***Je***(PCR) and ***Je***(PCO) but also significantly suppressed the increase of ***Ja***(O_2_-dependent) (**Table 1**). This indicated melatonin could play an important role in maintaining the capacity of PCR and PCO cycles to consume surplus photosynthetic electrons, thus decreasing the alternative electron flux depending on the partial pressure of O_2_. Similar results have been reported previously. Thus, Wei [56] found that *Pet F*, an electron transporter gene, and *rbcS, GAPC1, GAPCP-2*, genes in the Calvin cycle were upregulated by melatonin in salt-stressed soybean plants. Melatonin also helped maintain the photosynthesis rate of apple leaves under salt stress and senescence (Li et al., 2012; Wang et al., 2012). The sharp increase in ***Je***(PCO) suggests that photorespiration may play a key role in melatonin regulation. Our results indicate that melatonin helps maintain the PCR and PCO cycles and lessen the alternative electron flows depending on the O_2_ partial pressure which is closely related the production of ROS.

## Conclusion

We found that chilling stress led to serious chloroplast damage due to over-accumulation of ROS. Exogenous melatonin increased the chilling tolerance of chloroplast in cucumber seedlings by enhancing the AsA–GSH cycle to increase the ROS scavenging capacity and by adjusting photosynthetic electron flux so as to suppress the production of ROS.

## Author Contributions

ZZ, HZ, and YW designed the experiments, HZ, LY, XZ, JY, and JW carried out the experiments, HZ, YW, and KC contributed to the writing of the manuscript.

## Conflict of Interest Statement

The authors declare that the research was conducted in the absence of any commercial or financial relationships that could be construed as a potential conflict of interest.

The reviewer QW and handling Editor declared their shared affiliation, and the handling Editor states that the process nevertheless met the standards of a fair and objective review.

## Supplementary Material

**FIGURE S1 | Effects of exogenous melatonin on superoxide dismutase (SOD) activity in chloroplast under chilling stress.** *Control* (28/18°C); *Control + Melatonin* (200 μM melatonin, 28/18°C); *Chilling* (15/8°C); *Chilling + Melatonin* (200 μM melatonin, 15/8°C). Data are means ± SD of three replicates.
