# Supplementary material for: Melatonin Increases the Chilling Tolerance of Chloroplast in Cucumber Seedlings by Regulating Photosynthetic Electron Flux and the Ascorbate-Glutathione Cycle
Source: Front Plant Sci. 2016 Dec 6;7:1814. doi: 10.3389/fpls.2016.01814 (PMC5138187; doi:10.3389/fpls.2016.01814)
Supplement: Supplementary file 1 [file Table_1.DOCX]

| Gene | NCBI Accession | Primer Sequence (5’-3’) |
| --- | --- | --- |
| *actin* | AB010922.1 | F: ATGGCCGATGCCGAGGATATR  R: TAGGAGCATCATCACCAGCAAAAC |
| *CsCu-ZnSOD* | XM_004145720.2 | F: CTCCATTTTCAATCTCTCATTATCC  R: ATAGAAGTGATTGTGCGGCCATAG |
| *CsFe-SOD* | XM_011661610.1 | F: TACACTGAACCTGAGTTCAACAAC  R: ACGTTCTCTGAGAAACCAAACAGA |
| *CsAPX* | XM_004149751.2 | F: CTGCTACTGTTTTTGGAACCGCCG  R: GCGGAGGAGAGGAAACGAGTAGTT |
| *CsMDHAR* | XM_011656419.1 | F: ACAGCCTTCTTCTGTTGCCTTCAG  R: CTCTATTGTCGTTGGCGAAATCCG |
| *CsDHAR* | XM_004145807.2 | F: ATGTCGGGCTCCAGAATCCAACCA |
|  |  | R: AAAGCGAGGAATTGGAAGGAAGGT |
| *CsGR* | XM_011660964.1 | F: GTCCGATAGTGCTGGAGGTGTTGG |
|  |  | R: CCATCCAAAGCCATGACTCTCTTC |

**Table. S1** Sequences for primers used in quantitative real-time RT-PCR.
